# Biogenic Amines in Insect Antennae

**DOI:** 10.3389/fnsys.2017.00045

**Published:** 2017-06-28

**Authors:** Marianna I. Zhukovskaya, Andrey D. Polyanovsky

**Affiliations:** Laboratory of Evolution of Sense Organs, Sechenov Institute of Evolutionary Biochemistry and Physiology, Russian Academy of SciencesSaint Petersburg, Russia

**Keywords:** insect, antenna, sensory plasticity, octopamine, tyramine, serotonin, dopamine

## Abstract

Insect antenna is a multisensory organ, each modality of which can be modulated by biogenic amines. Octopamine (OA) and its metabolic precursor tyramine (TA) affect activity of antennal olfactory receptor neurons. There is some evidence that dopamine (DA) modulates gustatory neurons. Serotonin can serve as a neurotransmitter in some afferent mechanosensory neurons and both as a neurotransmitter and neurohormone in efferent fibers targeted at the antennal vessel and mechanosensory organs. As a neurohormone, serotonin affects the generation of the transepithelial potential by sensillar accessory cells. Other possible targets of biogenic amines in insect antennae are hygro- and thermosensory neurons and epithelial cells. We suggest that the insect antenna is partially autonomous in the sense that biologically active substances entering its hemolymph may exert their effects and be cleared from this compartment without affecting other body parts.

## Introduction

**Figure 1:**
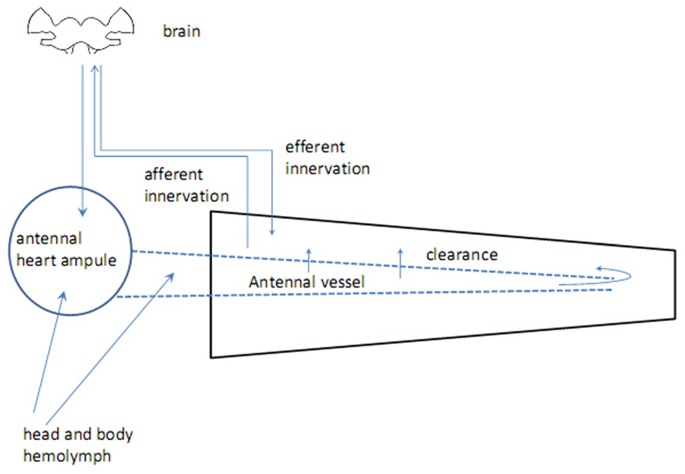
Origins and routs of biogenic amines in antenna.

**Figure 2:**
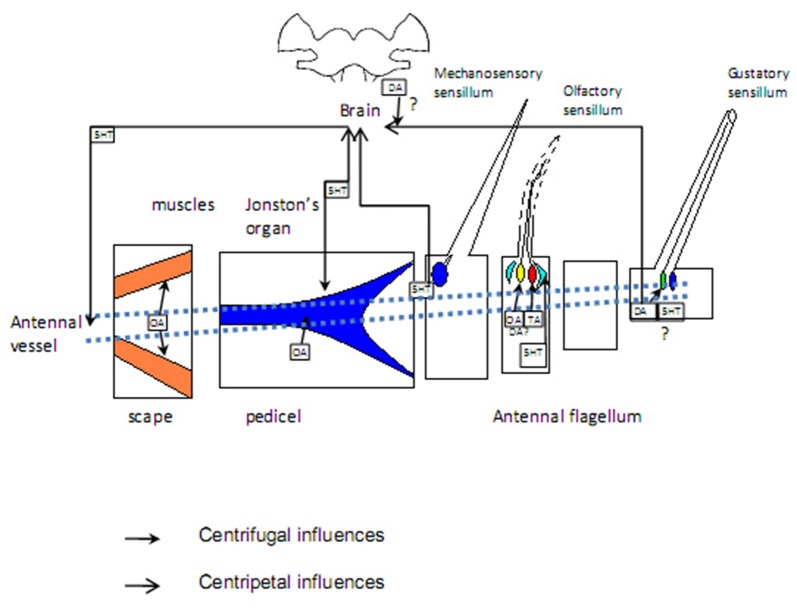
Targets of aminergic regulation: sensilla of different modality, muscles in basal segments, antennal vessels. Sources of biogenic amines: sensory neurons. Question marks denote unresolved issues. Blue: mechanosensory structures; green: gustatory cells; yellow and red: olfactory cells responding to different odors; light blue: accessory cells; orange: muscles. See the text for more information.

Insect antennae are complex sensory appendages engaged in acquiring information from different mechanical, gustatory and olfactory as well as thermal and humidity cues (Altner et al., 1977). The antenna consists of two basal segments having muscles, which control antennal movements, and flagellum devoid of muscles but bearing sensilla, miniature sensory organs. The antenna is supplied by oxygen through the trachea, originating from the spiracles which are positioned laterally in the thoracic and abdominal segments (Newport, 1836; Yadav, 2003). Haemolymph flows through the antennal vessel pumped by the antennal heart, a circulatory organ found in a handful of insect species (Pass, 2000; Pass et al., 2006). The proximal part of the antennal vessel shows features of the ion-transporting function (Pawlowa, 1895). Hemolymph spills from the vessel into the antennal hemolymphatic space through the openings in the vessel walls, called ostia, and the distal pore (Kapitskii, 1984; Pass et al., 2006; Boppana and Hillyer, 2014). Hormones and other biologically active substances are delivered to the antennal lumen from the two main sources—body hemolymph and secretion from nerve terminals in the wall of the antennal heart (Beattie, 1976; Figure 1). No centrifugal axonal processes were found in the antennal flagella other than those coming from tachykinin-reactive cells in the mosquito *Culex salinarius*, which form axo-dendritic synapses with sensory neurons (Meola and Sittertz-Bhatkar, 2002). The neurohemal area in the antennal heart ampulla of the cockroach* Periplaneta americana* releases octopamine (OA) into the antennal hemolymph under control of dorsal unpaired median (DUM) neurons, originating from the suboesophageal ganglion (Pass et al., 1988). The possible targets for OA are: (1) sensory receptor organs—sensilla, tuned to olfactory, gustatory and various mechanical cues as well as to humidity and temperature; and (2) non-sensory tissues including the ion-transporting epithelium in the antennal vessel (Pass, 1985) and hypoderm (Figure 2). Considerable length of antennae suggests the possibility of mainly local humoral modulation, since active substances contained in the hemolymph have enough time to exert their effect and be cleared out before returning to the head and body hemocoel (Figure 1).

The tremendous ability of insects to prosper owes, in part, their behavioral plasticity in response to environmental cues. Perception of pheromones and non-pheromone odors by an insect changes depending on various factors, such as age (Takasu and Lewis, 1996; Mechaber et al., 2002; Bohbot et al., 2013), physiological state (Blaney et al., 1986; Anton et al., 2007; Evenden and Gries, 2008), previous experience (Anton et al., 2011; Minoli et al., 2012; Riffell and Hildebrand, 2016), sensory surrounding (Yang et al., 2004; Zhao et al., 2013; Deisig et al., 2014) and circadian rhythmicity (Linn and Roelofs, 1986; Saifullah and Page, 2009; Schendzielorz et al., 2012).

During a long time, plasticity of behavioral responses was attributed to neuromodulation in the central nervous system rather than in antennal sensillae (Roelofs, 1995). However, after the study by von Nickisch-Rosenegk et al. (1996), showing the presence of biogenic amine receptors in the moth antenna, it is accepted now that all levels, from sensory input to motor output, involved in the organization of olfaction-guided behavior are under neurohumoral control (Anton et al., 2007).

All receptors of biogenic amines revealed in insects thus far represent membrane-bound G protein-coupled receptors (GPCRs), triggering different signaling cascades, which lead to a rise or fall in the cAMP level and Ca^2+^ release (Blenau and Baumann, 2001; Beggs et al., 2011; Ohta and Ozoe, 2014; Vleugels et al., 2015). The cross-talk between their signaling pathways and the intracellular biochemical machinery in antennal tissues is attracting attention of researchers (Flecke and Stengl, 2009; Flecke et al., 2010; Chen and Luetje, 2014), but is not studied in detail.

This review addresses those peripheral effects of biogenic amines—OA, tyramine (TA), serotonin (5-HT), and dopamine (DA)—that are confined to the antenna, the major sensory organ in insects.

## Octopamine

### Olfactory Reception

OA, topically applied or injected into an insect body, evokes pronounced changes in olfactory responses, both behavioral (Linn and Roelofs, 1986; Linn et al., 1992; Zhukovskaya, 2008) and electrophysiological (Pophof, 2000, 2002; Grosmaitre et al., 2001; Kapitsky and Zhukovskaya, 2001; Zhukovskaya and Kapitsky, 2006; Flecke and Stengl, 2009; Zhukovskaya, 2012). Besides, OA increases the spontaneous activity of pheromone-sensitive olfactory receptor neurons (ORNs) in some insect preparations (Grosmaitre et al., 2001; Zhukovskaya and Kapitsky, 2006; Flecke and Stengl, 2009; Stengl, 2010). In general, OA enhances behavioral responses to attractants (Linn and Roelofs, 1986; Zhukovskaya, 2008; Ma et al., 2015), improves nestmate recognition in ants (Vander Meer et al., 2008), and sometimes changes the valence of an odor, making neutral odor attractive and repellent odor neutral, as shown previously (Zhukovskaya, 2012).

Although these data shed some light on the role of octopaminergic regulation in insect olfaction, two issues were left unresolved. First, endogenous OA release can be induced by experimental manipulatons. For example, handling alone evokes a 3-fold rise in the OA level in the *P. americana* antennal heart, which supplies the antenna with hemolymph (Möbius and Penzlin, 1993). Besides, injections of agonists as well as antagonists of OA and TA induce a similar rise in displacement grooming suggested to be a dearousing behavior due to stress-induced changes in the OA level (Fussnecker et al., 2006). Second, response modulation, usually attributed to a direct effect of OA, may result from the indirect effect of other biologically active molecules released by OA. Outside the antennae, OA significantly changes the production of juvenile hormone and 20-hydroxyecdysone in cockroaches and flies (Downer et al., 1984; Gruntenko et al., 2007) and adipokinetic hormone in crickets (Orchard et al., 1983), which affect multiple targets throughout the insect body.

Upregulation of single cell olfactory responses coincides with an OA-induced decrease in the electroantennogram (EAG) amplitude. However, it is noteworthy that changes in EAG under the influence of OA and other humoral factors should be interpreted with caution, since EAG is a complex signal rather than just a voltage drop caused by arithmetical summation of leak currents from ORNs. EAG strongly depends on the electrical resistance of the antennal tissue as well as on the position of recording electrodes relative to the responding sensilla (Nagai, 1981; White, 1991; Kapitskii and Gribakin, 1992). Data reported by Dolzer et al. (2001) showed a dose-dependent decrease in the resistance of the sensillum preparation in the moth* Manduca sexta* under the impact of OA. The simultaneous OA-induced decrease in the EAG amplitude and increase in the action potential frequency led us to suggest that OA causes a depolarization in the ORN resting potential (Kapitsky and Zhukovskaya, 2001). As a result, the reduced ORN membrane potential requires smaller changes to reach the threshold potential level, at which action potentials are triggered, creating smaller currents recorded as EAG after the summation of these miniature leaks from responding ORNs along the antennal length. At the same time, the depolarized membrane is less stable, triggering more spikes in response to the odor stimulus. Recently, this concept has gained further support (Flecke and Stengl, 2009; Stengl, 2010).

Taking the advantage of the fact that the antennal flagellum is devoid of OA-producing structures, we performed single sensillum recording from the preparations of the isolated cockroach antennal flagellum (Kapitsky and Zhukovskaya, 2001; Zhukovskaya and Kapitsky, 2006) perfused with OA and OA-free saline. This approach allowed us both to administer and wash out OA as well as to control other hemolymp-born substances that bathe proximal parts of ORNs. The increase in the spiking rate in sex-specific male cockroach sensilla in response to pheromone and its background activity proved unequivocally that response modulation in the pheromone-sensitive ORNs results from the direct effect of OA. This finding was later supported in the other cockroach species, *Rhyparobia (Leucophaea) maderae* (Schendzielorz et al., 2012), using perfused sensillum preparations. OA-dependent local release of tachykinin inside the sensillum, as suggested by Jung et al. (2013), provides further downstream regulation of cells in the olfactory sensilla, possibly affecting EAG responses to odors.

Peripheral responses to general odorants may or may not be under OA control (Pophof, 2002; Zhukovskaya, 2012). Our data suggest that some of the hexanol-1-sensitive sensilla, morphologically and physiologically differing from the sex-pheromone sensitive ones (Schaller, 1978; Fujimura et al., 1991), are under OA control in adult *P. americana* males, while other sensilla are not affected (Zhukovskaya, 2012). To ascertain if the olfactory sensillum is modulated by OA via its effect on accessory cells, which partially create a driving force for the receptor current in response to odorants (Kaissling, 1987) and control the composition of the sensillum lymph (Thurm and Küppers, 1980; Keil, 1999), or each ORN is affected independently, we took the advantage of the fact that *P. americana* pheromone-sensitive sensilla houses both pheromone-sensitive and general odorant-sensitive “eucalyptol” cells. OA application enhanced firing responses of this type of sensilla to both pheromone components, periplanones A and B, but did not affect responses to eucalyptol (Zhukovskaya and Kapitsky, 2006; Zhukovskaya, 2012). Thus, in contrast to the cells responding to pheromone components and controlled by OA, the cell responding to general odorant is not OA-controlled. These data provide evidence that receptor cells inside the same sensillum, at least in some cases, are controlled independently via biogenic amine receptors on the ORN membrane. It is important to note that all the tested odorants, namely, pheromone components and plant-derived odorants, eucalyptol and hexanol, showed a decrease in EAG under the effect of OA. We did not detect significant changes in firing responses to eucalyptol, but cannot rule out that other receptor cells in other types of sensilla respond to this odor differently in the presence or absence of OA. Another possible explanation of the uniform EAG decrease under the effect of OA in response to all tested odors is a change in the electrical resistance of non-sensory antennal tissues, such as hemolymph, epithelium or cuticle, which contributes to the cumulative resistance of the antennal preparation. It appears that OA release into antennal hemolymph switches the mode of its functioning, altering the antennal sensitivity to a particular set of pheromone components and environmental odors in order to better conform the specific needs of the animal.

Coupling of olfactory sensitivity modulation by OA with circadian rythmicity was initially found in the cabbage looper moth *Trichoplusia ni* (Linn and Roelofs, 1986; Linn et al., 1992). Later, OA modulation of olfactory sensitivity in the antenna of *M. sexta* was found to be linked to the circadian rhythmicity in pheromone reception through cAMP-dependent disadaptation in receptor cells (Flecke and Stengl, 2009; Flecke et al., 2010; Stengl, 2010). The antennal OA receptor cloned in the *M. sexta* shares high sequence similarity with other insect α-adrenergic-like OA receptors and increases both cAMP and Ca^2+^ intracellular concentration in response to an agonist (Dacks et al., 2006). Ca^2+^ and cAMP levels altered due to OA-induced signal transduction are supposed to act through metabotropic activation of Orco, an odorant receptor coreceptor protein, leading to changes in ORN sensitivity (Getahun et al., 2013; Stengl and Funk, 2013).

Octopaminergic modulation in the antenna can be enhanced not only by the elevation of the OA level, but also by upregulation of OA receptors in the antenna of honey bee workers, as shown using the Real-time qPCR technique (McQuillan et al., 2012). Thus, expression of OA receptors (AmOA1) in the antenna was found to be higher in young nurses as compared to pollen foragers of the same age, corresponding to sensitivity to queen mandibular pheromone (QMP). Foragers in the bee colony are not attracted by QMP while the expression level of antennal AmOA1 is low (Vergoz et al., 2009). AmOA1 also belongs to the α-adrenegic-like OA receptor family that induces an oscillatory increase in the intracellular Ca^2+^ concentration under OA stimulation but only a slight elevation of the cAMP level (Grohmann et al., 2003).

### Other Targets of Octopamine in Insect Antenna

Muscles situated in the first two antennal segments, scape and pedicel (Chapman, 1998), control movements of the flagellum through motoneurons and modulating neurons, including octopaminergic DUM neurons, descending from the suboesophageal ganglion (Bräunig et al., 1990; Bauer and Gewecke, 1991; Baba and Comer, 2008; Figure 2). Stimulation of these cells as well as OA application attenuates slow and enhances fast contractions in cricket preparations (Allgäuer and Honegger, 1993), facilitating fast antennal movements during tracking a target.

The mechanosensory Johnston’s organ, responding to vibrations and low frequency sounds, was recently found to be modulated by OA, which shifts frequency tuning and is likely to allow mosquito males to track females by following their changing flight sound tones due to movement (Andrés et al., 2016). Interestingly, despite the fact that OA plays an important role in arousal and aggression, the threshold for mechanical stimulation of antennae, causing an aggressive response in male crickets, does not depend on OA (Rillich and Stevenson, 2015; Stevenson and Rillich, 2016). Thus, central rather than peripheral mechanosensory octopaminergic modulation is responsible for adjusting the level of aggression in response to stimulation of antennal mechanosensitive sensilla.

## Tyramine

TA is, on the one hand, an OA biosynthetic precursor, but on the other hand, it plays a distinctive role in an insect body. Since there were identified some TA-containing neurons devoid of OA, the specific role of TA as a neuroactive compound became evident (Nagaya et al., 2002). In fact, TA and OA are believed to be, in a sense, functionally antagonistic (Roeder et al., 2003; Roeder, 2005; Lange, 2009). For example, in contrast to attractive (pheromone) odors modulated by OA, behavioral responses to aversive (non-pheromone) odors are affected by TA because they were decreased in *hono*, the *Drosophila melanogaster* TA (TA/OA) receptor knockout (Kutsukake et al., 2000). However, it is preliminary to conclude that OA modulates pheromone-sensitive ORNs while TA affects general odorant-sensitive ORNs, because our above data on OA-upregulated responses to the non-pheromone repellent odor of hexanol (Zhukovskaya, 2008, 2012) indicate that responses to general odorants can be regulated by OA. It is also unlikely that there is a strict division of functions, when TA modulates responses to repellents whereas OA modulates attractants, because the valence of a particular odor can be changed by learning and other experience-based effects (McCall and Eaton, 2001; Saleh and Chittka, 2006; Anderson and Anton, 2014). X-gal staining of the *hono* gene product revealed about 10 most probable ORN candidates in the third antennal segment, the main olfactory organ in an adult fly, as well as in the larval dorsal olfactory organ. Although first lepidopteran OAR/TAR, identified in *B. mori* and *H. virescens*, were thought to be OAR (von Nickisch-Rosenegk et al., 1996), it has been demonstrated later that at least in *B. mori* OAR/TAR is two orders of magnitude more sensitive to TA than to OA and shows much higher affinity (by about 270 times) to TA than OA, representing, in fact, a TA receptor. TA activation of this receptor leads to G_i_ protein-mediated inactivation of adenylate cyclase and a reduction in intracellular cAMP levels (Ohta et al., 2003, 2004).

In honey bee antennae, downregulation of OA receptors Amoa1 and upregulation of TA receptors AmTAR1 were revealed during transition from QMP-sensitive nurses to plant odor-sensitive foragers as detected by a real-time quantitative PCR technique (McQuillan et al., 2012). Another TA receptor from the honey bee, AmTAR2, was shown to increase the intracellular cAMP level in the flpTM heterologous expression system (Reim et al., 2017), but so far is not found in insect antennae.

In the adult *M. brassicae*, TA receptor (MbraOAR/TAR) transcripts were detected both in pheromone- and general odor-sensitive antennal sensilla (Brigaud et al., 2009). TA not only affects ORNs in insect antennae, but itself can be synthesized by some of them (Figures 1, 2). Presumably, in the blowfly *Phormia regina* it acts in the antennal lobe neuropile through modulation of responses to aversive odor of d-limonene (Ishida and Ozaki, 2012). Since TA receptors mostly decrease while OA receptors increase the cAMP level (Ohta and Ozoe, 2014), their effects on cAMP-dependent intracellular events should be mutually opposite, but whether these receptors co-localize in the same cell is an open question.

Tryptamine, produced in plants as their defense reaction against insect herbivores, was found to be antagonistic to olfactory co-receptor Orco in the low micromolar range (Chen and Luetje, 2014), probably interacting with TA or OA binding sites.

## Serotonin

It is generally accepted that in insect antennal ORNs, the role of neurotransmitter is played by acetylcholine, although there are a few pieces of evidence deviating from this tenet. Serotonin-immunoreactive fibers were identified in the antennal nerve of *P. americana* (Salecker and Distler, 1990), projecting into antennal mechanosensory and contact chemosensory centers mainly in the deutocerebrum. Later, cell bodies of these sensory neurons were found in mechanosensory chaetic and scolopoidial sensilla in the scape, pedicel and first 15 flagellomeres. Moreover, efferent fibers were found within the scape, ramifying along the antennal vessel and inner margin of the epidermal layer without contacting them synaptically (Watanabe et al., 2014). Serotoninergic efferent fibers have also been identified in mosquitoe antennae, where they are targeted at the antennal flagellum and scolopidia of the Johnston’s organ (Siju et al., 2008; Andrés et al., 2016). Transcriptomic analysis revealed few putative 5-HT receptor proteins in the antennae of the mosquito* Anopheles gambiae* (Pitts et al., 2011), supporting the role of 5-HT as a neurohormone. 5-HT affects the transepithelial potential, generated by accessory cells in the olfactory sensillum and creating a driving force for the receptor current (Dolzer et al., 2001; Grosmaitre et al., 2001).

The direct effect of 5-HT on firing responses in the blowfly *Phormia regina* labellar gustatory receptor neurons during the specific stage of their ovarian maturation period indicates a peripheral modulation of gustatory receptor neurons. Exogenous 5-HT supply specifically increases the chemoreceptor sensitivity to sugar at the mature ovaries and post egg-laying stages (Solari et al., 2015). However, it is not clear whether antennal gustatory neurons are serotonin-modulated or the effect of 5-HT is labellum-specific and this issue should be a matter of future research (Figure 2).

## Dopamine

### Olfactory Reception

There are indications that DA can serve as a neurohormone, modulating odor responses. Expression of the DA receptor Amdop3 in the honey bee antenna was found to correlate with an age-dependent decrease in sensitivity of honey bee workers to the QMP component (Vergoz et al., 2009; McQuillan et al., 2012). The age-dependent decrease in pheromone sensitivity in the male black cutworm* Agrotis ipsilon* is also thought to be associated with DA signaling via the G protein-coupled DA/ecdysteroid receptor AipsDopEc, however, this effect is attributed to the brain level, since antennal expression was low and age-independent (Abrieux et al., 2013).

### Gustatory Reception

Most data on gustatory reception were obtained on flies, which bear short antennae unable to touch the substrate to perform gustatory function, whereas flies taste food using sensilla located on the labella. Gustatory plasticity, similarly to above-described olfactory plasticity, is achieved at different levels of sensory processing. Starvation (nutritional stress) causes changes in the sugar-sensitive gustatory receptor on the fly labellar sensilla due to increased expression of the Gr64a receptor gene (Nishimura et al., 2012). Since responses to nutritional stress in flies are accompanied by changes in biogenic amine levels (Gruntenko et al., 2005), it was logical to look for dopaminergic regulation in gustatory receptor neurons. DA receptors were found to enhance sucrose sensitivity under starvation in *Drosophila* sucrose-sensitive gustatory receptor cells (Inagaki et al., 2012). At the same time, bitter sensitive neurons decrease their output during OA and TA modulation (Inagaki et al., 2014; LeDue et al., 2016). In both cases, however, modulation occurs presynaptically on axonal terminals, projecting from the fly labellum to primary gustatory neuropile of the suboesophageal ganglion.

A majority of insects other than flies bear gustatory sensilla on antennae and are likely to use similar dopaminergic regulatory mechanisms. For example, unpaired H-cells with their bodies located in the suboesophageal ganglion of moths and orthopterans (Mesce et al., 2001) release DA that can be transported to antennae (Galizia and Rössler, 2010).

DA receptors were demonstrated to be expressed in honey bee antennae; moreover, changes in the expression level of one of them, Amdop1, corresponded to the transition from nursing to foraging (McQuillan et al., 2012). Since QMP contains some non-volatile components, DA appears to be a plausible modulator candidate in pheromone-sensing gustatory receptor neurons (Figure 2).

### Other Possible Targets of Aminergic Modulation in Antenna

The outer layer of the cuticle bears waxes or liquid cuticular hydrocarbons, the repertoire of which may be body part specific (Oppelt and Heinze, 2009; Bagnères and Blomquist, 2010). Our data suggest that the liquid coating of the cockroach antenna plays an important role in olfaction (Böröczky et al., 2013), providing, in concert with grooming, odorant cleanout from the antennal surface. It can be hypothesized that the cuticular lipid secretion is modulated via the neurohumoral (probably, aminergic) mechanism. Thermosensory neurons may also be modulated by the hemolymph-born molecules. No direct measurements of thermosensory neuronal responses in the presence of biogenic amines have been found in literature, but some clues on the possibility of the modulation can be found. For example, in the blood-feeding yellow fever mosquito *Aedes aegypti*, long-range perception of CO_2_ changes behavioral responses to a short-range thermal signal (McMeniman et al., 2014). The satiety level, interrelated with the 5-HT titer (Lange et al., 1989), influences the response to heat in the bug *Rhodnius prolixus* (Bodin et al., 2009). Previous experience changes behavioral responses to thermal stimulation in the worker ant *Camponotus rufipes* (Weidenmüller et al., 2009) and bumblebee *Bombus terrestris* (Westhus et al., 2013) in a way similar to that described for olfactory reception.

## Conclusions

The insect antenna is a multisensory organ, and each modality can be modulated by biogenic amines. It appears that insect antenna is partially autonomous in the sense that biologically active substances entering its hemolymph may exert their specific effects and be removed predominantly or even totally inside this compartment without affecting other body parts. OA increases activity of pheromone- and some, but not all, non-pheromone-sensitive antennal ORNs. There is some evidence that DA modulates gustatory receptor neurons. TA, a metabolic OA precursor, also modulates ORNs, usually in an antagonistic manner to OA, but it is unclear if TA and OA receptors are co-localized in receptor neurons. Serotonin can serve as a neurotransmitter in some afferent mechanosensory neurons and both as a neurotransmitter and neurohormone in efferent fibers targeted at the antennal vessel and mechanosensory organs. Aminergic modulation of thermo- and hygrosensory sensilla has not yet been demonstrated, and could potentially be another target for modulation. Functioning of non-sensory antennal tissues in the epithelium, tracheae and hemolympatic vessel may also be under humoral control, including aminergic.

## Author Contributions

MIZ, ADP: manuscript planning, MIZ: draft writing, ADP: editing.

## Conflict of Interest Statement

The authors declare that the research was conducted in the absence of any commercial or financial relationships that could be construed as a potential conflict of interest.
